# Clinical pharmacodynamic/exposure characterisation of the multikinase inhibitor ilorasertib (ABT-348) in a phase 1 dose-escalation trial

**DOI:** 10.1038/s41416-018-0020-2

**Published:** 2018-03-19

**Authors:** Michael L. Maitland, Sarina Piha-Paul, Gerald Falchook, Razelle Kurzrock, Ly Nguyen, Linda Janisch, Sanja Karovic, Mark McKee, Elizabeth Hoening, Shekman Wong, Wijith Munasinghe, Joann Palma, Cherrie Donawho, Guinan K. Lian, Peter Ansell, Mark J. Ratain, David Hong

**Affiliations:** 1 Department of Medicine, Section of Hematology/Oncology, University of Chicago, 5841 S Maryland Avenue, Chicago, IL 60637 USA; 2 Committee on Clinical Pharmacology and Pharmacogenomics, University of Chicago, 5841 S Maryland Avenue, Chicago, IL 60637 USA; 3 Comprehensive Cancer Center, University of Chicago, 5841 S Maryland Avenue, Chicago, IL 60637 USA; 4 Department of Investigational Cancer Therapeutics, The University of Texas, MD Anderson Cancer Center, 1400 Holcombe Boulevard, Unit 455, Faculty Center 8th Floor, Houston, TX 77030 USA; 5 Sarah Cannon Research Institute at HealthONE, Drug Development, 1800 N Williams Street Suite 300, Denver, CO 80218 USA; 6 Center for Personalized Cancer Therapy, Moores Cancer Center, The University of California San Diego, 3855 Health Sciences Drive, La Jolla, CA 92093 USA; 7 AbbVie Inc., 1 N Waukegan Road, North Chicago, IL 60064 USA; 8 Present Address: Inova Schar Cancer Institute, Inova Center for Personalized Health, and Virginia Commonwealth University, 3225 Gallows Road, Falls Church, VA 22037 USA

**Keywords:** Predictive markers, Tumour biomarkers

## Abstract

**Background:**

Ilorasertib (ABT-348) inhibits Aurora and VEGF receptor (VEGFR) kinases. Patients with advanced solid tumours participated in a phase 1 dose-escalation trial to profile the safety, tolerability, and pharmacokinetics of ilorasertib.

**Methods:**

Ilorasertib monotherapy was administered at 10–180 mg orally once daily (Arm I, *n* = 23), 40–340 mg orally twice daily (Arm II, *n* = 28), or 8–32 mg intravenously once daily (Arm III, *n* = 7), on days 1, 8, and 15 of each 28-day cycle.

**Results:**

Dose-limiting toxicities were predominantly related to VEGFR inhibition. The most frequent treatment-emergent adverse events ( > 30%) were: fatigue (48%), anorexia (34%), and hypertension (34%). Pharmacodynamic markers suggested that ilorasertib engaged VEGFR2 and Aurora B kinase, with the VEGFR2 effects reached at lower doses and exposures than Aurora inhibition effects. In Arm II, one basal cell carcinoma patient (40 mg twice daily (BID)) and one patient with adenocarcinoma of unknown primary site (230 mg BID) had partial responses.

**Conclusions:**

In patients with advanced solid tumours, ilorasertib treatment resulted in evidence of engagement of the intended targets and antitumour activity, but with maximum inhibition of VEGFR family kinases occurring at lower exposures than typically required for inhibition of Aurora B in tissue.

Clinical Trial Registration: NCT01110486

## Introduction

Since 2001, at least 27 small-molecule kinase inhibitors have been approved by the US Food and Drug Administration for indications in oncology.^1,2^ However, because of the multiplicity of targets of most kinase inhibitors, their development and safe clinical use continue to present challenges. Increasingly, cancer therapeutic development plans have incorporated concepts of the pharmacologic audit trail, a systematic serial assessment of an anticancer compound’s pharmacology as it proceeds from preclinical through clinical development.^3^ Updated methodologic practices^4^ include a more complete evaluation of pharmacokinetics and systemic pharmacodynamics in human subjects to match the intensive assessment of pharmacodynamics in tumours and tumour models. Especially for kinase inhibitors, these assessments can redirect development of individual compounds, and sharing the results can inform development of other compounds with similar targets.

Commonly, kinase inhibitors are adenosine triphosphate (ATP) mimetics chosen through in vitro assays because of their properties of relative selectivity for a specific intended set of target kinases. Multiple methods have been developed to characterise properties of these compounds in cells and to predict the effects of these drugs on tissues, patients, and populations. For example, high-throughput screening of compound libraries against large numbers of human kinases followed by comprehensive analysis of interaction patterns has provided valuable information about the selectivity of kinase inhibitors.^5–7^ In addition, computational large-scale integration of disease gene expression signatures with compound effect signatures may be a useful strategy to predict compound–disease relationships.^8^ Integrative computational approaches can also be used to predict the effect of drugs on immune function and minimise side effects.^9^ Nevertheless, activity predictions, while useful, are approximations, and multiple factors determine how these agents will affect patients. It therefore becomes important to re-characterise these drugs during the conduct of first-in-human and other early phase trials. A change in a compound’s development plan and design of subsequent studies should be based on this initial in-human re-characterisation.

Ilorasertib (ABT-348) is an ATP-mimetic kinase inhibitor that was selected for clinical development on the basis of its dual-inhibition properties of Aurora and vascular endothelial growth factor receptor (VEGFR) kinase signalling pathways.^10^ Aurora kinases are key regulators of mitosis that are overexpressed in many cancer types and have been identified as candidate drug targets for cancer therapy.^11–13^ In enzyme assays to determine kinase inhibitory activity, ilorasertib showed in vitro potency against a panel of cancer-related kinases (Table 1,^10,14^ with greater potency for inhibiting binding and cellular autophosphorylation of Aurora B and C, compared with Aurora A. In addition, ilorasertib inhibited VEGFR and platelet-derived growth factor receptor (PDGFR) families of kinases in the low nanomolar range. As with inhibition of Aurora kinase activity, inhibition of VEGFR/PDGFR kinases in biochemical assays correlated with inhibition of cellular autophosphorylation of targeted kinases for VEGFR2, fms-like tyrosine kinase 3 (FLT-3), colony-stimulating factor 1 receptor (CSF-1R), c-KIT, and PDGFR-α and PDGFR-β.^10^ Evidence of the in vivo activity of ilorasertib was confirmed in xenograft models. Concurrently, the sponsor began this solid tumour study and a separate phase 1 dose-escalation trial in patients with haematologic malignancies. The latter trial completed accrual and achieved its primary endpoints: safety, pharmacokinetics, and preliminary antitumour activity.^15^

**Table 1:** Ilorasertib enzyme and cellular pharmacodynamics potency

Kinase	Biochemical IC_50_, nM^a^ (95% confidence limits)	Cellular PD marker IC_50_, nM (95% confidence limits)
Aurora A	120 (117–123)	189 (153–233)^b^
Aurora B	7 (2–14)	13 (5–27)^b^
		21 (11–42)^c^
Aurora B (Y156H)^d^	12 (11–17)	ND
Aurora C	1 (1–2)	13 (5–27)^b^
VEGFR1	1 (0.6–2)	0.3 (0.1–0.4)^e^
VEGFR2	2 (1–3)	5 (4–7)^e^
VEGFR3	43 (18–93)	2 (0.1–23)^e^
FLT-3	1 (0.9–2)	2 (2–3)^e^
CSF-1R	3 (2–4)	3 (0.8–8)^e^
c-KIT	20 (6–25)	45 (33–64)^e^
PDGFR-α	11 (6–21)	16 (6–19)^e^
PDGFR-β	13 (1–46)	11 (4–28)^e^

*ATP*adenosine triphosphate, *CSF-1R* colony-stimulating factor 1 receptor, *FLT-3*fms-like tyrosine kinase 3, *IC*_*50*_ half maximal inhibitory concentration, *ND*not determined, *PD* pharmacodynamic, *PDGFR*platelet-derived growth factor receptor, *VEGFR* vascular endothelial growth factor receptor.

^a^Enzyme assays were conducted in homogeneous time-resolved fluorescence format using 1 mM ATP.^14^

^b^Aurora A, B, and C autophosphorylation was performed in nocodazole-arrested HeLa cells by western analysis using phospho-A, phospho-B, and phospho-C-specific antibodies.

^c^Phosphorylation of histone H3.

^d^Aurora B kinase Y156H mutant.

^e^Cellular phosphorylation assays for VEGFR2, CSF-1R, KIT, PDGFR-α,^14^ and PDGFR-β were ligand stimulated for 5 to 20 min for optimal phosphorylation depending on receptor type. FLT-3 was constitutively phosphorylated in SEM cells; inhibition was determined after 60 min of exposure to the compound. VEGFR1 activity was determined in BaF3 cells expressing the TEL:VEGFR1 catalytic domain fusion using proliferation as a surrogate readout.

Reproduced by kind permission of the American Society for Pharmacology and Experimental Therapeutics, from Glaser et al.^10^

We enrolled patients with diverse advanced solid tumours in a phase 1 dose-escalation trial of ilorasertib. In the early dose cohorts of the trial, adverse effects typically attributed to VEGFR inhibition were observed with greater frequency than those typically attributed to Aurora kinase inhibition. The subsequent analysis of pharmacodynamic biomarkers reported here provided evidence that maximum inhibition of VEGFR2 in the systemic vasculature occurs at lower exposures than required to inhibit Aurora kinase in tissue—concordant with the observed relationship with VEGFR2-inhibition-related adverse events. We employed measurement of fit-for-purpose clinical, plasma, and skin biomarkers to inform the future development of this single compound with multiple intended kinase targets.

## Materials and methods

### Study design and objectives

This was a phase 1 multicentre, open-label, dose-escalation study (NCT01110486) to evaluate the safety, pharmacokinetics, and pharmacodynamics of ilorasertib in patients with advanced solid tumours. Patients were enrolled from March 2010 to May 2012. The study was to be conducted in two distinct stages, with the first involving a dose-escalation cohort and the second an expanded safety cohort. However, due to the discontinuation of this programme, no patients were enrolled in the second stage. Dose escalation proceeded using an adaptation of the continual reassessment method^16,17^ with the objectives of defining dose-limiting toxicities (DLTs) and subsequent maximum-tolerated dose (MTD). The study enrolled patients into five treatment arms (I–V); Arms IV and V were combination arms in which ilorasertib was combined with carboplatin or docetaxel, respectively. Herein, we report the findings for the three monotherapy arms. All patients in Arms I, II, and III received ilorasertib monotherapy on days 1, 8, and 15 of each 28-day cycle. Ilorasertib was administered orally (PO) once daily (QD) in Arm I, PO twice daily (BID) in Arm II, and via 2-h intravenous (i.v.) infusion in Arm III. In Arm II, dosing was separated by a 6-h interval with the total dose divided into two equal daily doses. In all treatment arms, patients continued ilorasertib treatment until disease progression or toxicity related to ilorasertib that failed to resolve to grade ≤ 2 within 3 weeks. The primary objectives of the study were to determine the safety profile, evaluate the pharmacokinetics, and determine the MTD of ilorasertib monotherapy. Secondary objectives included characterisation of clinical pharmacodynamics of ilorasertib (biomarker analyses) and preliminary efficacy evaluation. All preliminary efficacy variables were exploratory in nature. The study received institutional review board approval and was conducted in accordance with International Conference on Harmonisation Good Clinical Practice guidelines, and ethical principles that have their origin in the Declaration of Helsinki. All patients provided their written informed consent.

### Patient eligibility criteria

Patients aged ≥ 18 years with histologically confirmed locally advanced or metastatic solid tumours that were refractory to standard therapy were eligible. In addition, patients were required to have an Eastern Cooperative Oncology Group (ECOG) performance status of 0–2, adequate renal function (serum creatinine ≤ 1.5× the upper limit of normal (ULN) and either estimated creatinine clearance ≥ 50 mL/min as per Cockcroft-Gault formula or ≥ 50 mL/min based on 24-h urinalysis), liver function (serum bilirubin < 2× ULN and aspartate aminotransferase and alanine aminotransferase ≤2.5× ULN), and bone marrow function (absolute neutrophil count ≥ 1,500/mm^3^, platelets ≥ 100,000/mm^3^, and haemoglobin ≥ 9.0 g/dL), QTc interval < 500 ms on electrocardiogram, and left ventricular ejection fraction >50%.

*Patients with any of the following were excluded*: active central nervous system involvement including untreated brain or meningeal metastases, although patients with treated brain metastases that were radiographically or clinically stable for ≥4 weeks after therapy and had no evidence of cavitation or haemorrhage were eligible, provided they were asymptomatic and did not require corticosteroids; prior anticancer therapy within the past 21 days or five half-lives (whichever was shorter); any grade ≥ 2 unresolved toxicities from prior anticancer therapy; major surgery within past 28 days; symptomatic or persistent, uncontrolled hypertension; grade > 1 proteinuria; receiving anticoagulation therapy; or the patient had any medical condition, uncontrolled, or otherwise, which in the opinion of the investigator placed the patient at unacceptably high risk for toxicities or would limit compliance with study requirements. Concomitant use of CYP3A4 inhibitors and inducers was prohibited.

### Dose and treatment schedule

Dose escalation was first conducted in Arm I and subsequently in Arms II and III. For the three arms, dosing began in a cohort of three patients, with a doubling of the dose in subsequent dose cohorts until a DLT or a grade ≥ 2 toxicity attributable to ilorasertib occurred. The pre-specified DLT criteria included any of the following events graded according to the Common Terminology Criteria for Adverse Events (CTCAE) version 4.0 considered ‘possibly’ or ‘probably’ related to the administration of ilorasertib in cycle 1: grade 4 absolute neutrophil count (ANC) decrease for >7 days or grade ≥ 3 ANC decrease with fever; grade 4 platelet count decrease; grade ≥ 3 proteinuria; grade 4 infusion reaction; grade ≥ 3 asthenia/fatigue; grade 3 nausea/vomiting for ≥48 h or diarrhoea for ≥72 h despite maximum supportive care; grade ≥ 2 neurotoxicity; unexpected grade 2 toxicity which required dose modification or delay of ≥1 week. Given the known adverse effects of VEGFR2 inhibition, specific criteria for hypertension were included: >150/100 mm Hg despite 2 weeks of clinically appropriate intervention, or severe hypertension at any blood pressure reading defined as systolic >200 mm Hg or diastolic > 110 mm Hg, or symptomatic hypertension at any reading; and for decline in left ventricular ejection fraction included: absolute decline >20% from baseline and/or a reduction to <39%. Except for the above categories, all grade ≥ 3 adverse events (AEs) without a definitive alternative etiology were also considered DLTs. AEs detected or worsened in cycle 2 and beyond could be incorporated into dose-escalation decisions at discretion of the investigators and study team.

Once the first cohorts of three patients completed dosing in cycle 1, additional cohorts of a single patient at a time were treated at the new dose level in each arm, provided there were no DLTs or treatment-related toxicities exceeding grade 1. Once a grade ≥ 2 toxicity attributable to ilorasertib occurred at a given dose level, the current and all subsequent cohorts were to increase from one to three patients, and dose escalations were limited to a maximum of 50%, with specific dose levels determined by an adaptive continual reassessment method.^18^ The MTD was to be continually re-estimated by a logistic regression model as the dose at which the predicted DLT rate would be 30%. For the three arms, dose-escalation decisions were made after the patient(s) in a cohort completed dosing in cycle 1.

In Arm I, dosing commenced at 10 mg PO QD and was escalated to 180 mg PO QD. This initial starting dose was calculated using 1/6 of the highest non-severely toxic dose in dogs converted to human equivalent dose, using body surface area scaling and assuming a 60-kg human. Because of uncertainty of food effects on pharmacokinetics and the once-weekly schedule, treatment was administered in a fasted state. The 180-mg dose met protocol criteria for exceeding the MTD (i.e., two patients experienced DLTs) and was de-escalated to 140 mg, at which no further DLTs were reported. Enrolment to Arm I was discontinued, on the basis of the protocol-specified continual reassessment method on which MTD was to be determined. Nevertheless, cumulative evidence from the other arms of this study and the haematologic malignancies study^15^ suggested that DLT criteria did not accurately identify the MTD in Arm I. In phase I studies of ilorasertib in both solid tumours and haematologic malignancies, an alternate schedule of administration was evaluated, in which the once-weekly dose was divided into two consecutive daily administrations on a weekly basis. This schedule was selected due to the anticipated lower maximum observed plasma concentration (*C*_max_) and greater total exposure, and the possibility that tolerability would be improved under these conditions. Dose escalation proceeded in Arm II, starting at 40 mg PO BID, and stopped at the 340-mg PO BID dose, since this dose met the protocol criteria for exceeding the MTD. The first dose was administered in the fasted state. The second dose of the treatment day was administered at least 4 h after a small meal. In Arm III, dosing commenced at 8 mg i.v. over ~2 h QD and was escalated to 32 mg i.v. QD. Dosing at 32 mg i.v. QD resulted in grade 2 and 3 toxicities (not DLTs). At this point in the trial, data from the haematologic malignancies study intravenous dosing arm enabled an estimate of the absolute oral bioavailability for doses above 80 mg PO to be 12% and further enrolment in the intravenous arm of this solid tumour study was discontinued.

### Assessments

Safety was assessed on the basis of AEs, physical examination, vital signs, electrocardiograms, multiple-gated acquisition scans, and clinical laboratory test assessments. AEs were graded using National Cancer Institute CTCAE version 4.0.

Pharmacokinetics of ilorasertib were evaluated in the dose-escalation cohort on blood samples that were collected on cycle 1 days 1 and 15. Parameters determined: *C*_max_, time to *C*_max_ (*T*_max_), terminal phase elimination half-life (*t*_1/2_), clearance, apparent oral clearance (CL/F), and area under the plasma concentration-time curve from time 0 to time of last measurable concentration (AUC_t_) and from time 0 to infinity (AUC_∞_). For Arm I, blood samples were collected prior to dosing and at 0.5, 1, 2, 3, 4, 6, 8, 10 or 12, and 24 h after dosing on cycle 1 days 1 and 15; of note, for institutions not able to collect the 10- or 12-h post-dose sample, the 24-h sample was replaced with 22-h and 28-h post-dose samples. In Arm II, for the first dose, blood samples were collected pre-dose and at 1, 2, 3, and 4 h post-dose on cycle 1 days 1 and 15; for the second dose, samples were collected pre-dose and at 1, 2, 3, 4, 18, and 24 h after second dose on cycle 1 days 1 and 15. In Arm III, blood samples were collected prior to infusion, at 1 h 55 min (just before end of infusion), and at 0.5, 1, 2, 4, 6, 8, 10, and 24 h after end of infusion on cycle 1 days 1 and 15; here also, for institutions not able to collect the 8-h or 10-h after-end-of-infusion samples, the 24-h sample was replaced with 22-h and 28-h post-dose samples.

The liquid chromatography (LC)-mass spectrometry (MS)/MS method was developed and validated in the Bioanalysis Department of AbbVie. Fifty microliter of human plasma (K_2_EDTA as anticoagulant) was fortified with 300 µL of deuterium-stable labelled internal standard and mixed well. A HybridSPE precipitation plate (50 mg/96 wells, Supelco Analytical, Munich, Germany) was used to directly load and elute (under slight vacuum) the fortified samples into a clean collection plate. Samples were dried before reconstitution with 100 µL of 20/80 (v/v) acetonitrile/water, and injected for LC-MS/MS analysis. A Halo C8 (Wilmington, DE, USA; 2.7 µm, 2.1 × 30 mm) was used on an Agilent 1290 Infinity UPLC pump (Agilent Technologies, Santa Clara, CA) to carry out the separation. An isocratic condition with 25/75/0.1 (v/v/v) acetonitrile/water/98% formic acid was used as the mobile phase. The flow rate was set to be 0.6 mL/min. An MPS 3 C (Gerstel, Mülheim an der Ruhr, Germany) autosampler was used to introduce samples onto the UPLC system. An API 4000 mass spectrometer (Applied Biosystems, Foster City, CA) was used in the multiple reactions monitoring mode for the detection and quantitation of analyte.

The method was validated per US Food and Drug Administration guidance and internal standard operating procedures. The mean bias of lower limit of quantitation was –1.8%, with a coefficient of variance of 3%. Inter-run bias of quality controls ranged from –0.6 to 8.9%, with coefficient of variance < 7.6%. All plasma lots tested for matrix effect met acceptance criteria. Total analytic recovery ranged from 62.7 to 66.4% for the analyte, with stability demonstrated for at least five freeze/thaw cycles and 24 h at room temperature, and for at least 634 days when stored at –20° C. Post-extraction stability (autosampler and batch storage) was established for at least 72 h in an autosampler set at 10° C.

Pharmacodynamic analyses were conducted to better clarify the relationships between exposure parameters and extent of engagement of the intended therapeutic targets. Biomarkers to assess ilorasertib-mediated inhibition of both VEGFR and Aurora kinase pathways were measured.

To determine whether VEGF signalling was inhibited by ilorasertib, placental growth factor (PlGF) was assessed in Arm I at baseline and 24 h post-dosing. Plasma concentrations of PlGF were determined using an automated ARCHITECT system that utilised a patented chemiluminescent detection technology called CHEMIFLEX (Abbott Diagnostics, Abbott Park, IL). This method is based on a robust, well-validated plasma assay in clinical trials.^19^ In all study arms, diastolic blood pressure (DBP) measurements were obtained by trained observers, consistent with American Heart Association guidelines.^20^

To assess inhibition of Aurora kinase B signalling during ilorasertib treatment, 3-mm skin punch biopsies were collected from patients prior to and following ilorasertib exposure (dosing at 8 mg i.v. and 120–680 mg PO (60‒340 mg PO BID)). Biopsies were formalin fixed, paraffin embedded, and the levels of phosphohistone H3 (pHH3), a direct substrate of Aurora kinase B and therefore useful for assessing Aurora kinase B activity, were measured by immunohistochemistry using anti-pHH3, 1:100 (Cell Signaling #9701, Beverly, MA). In brief, after staining, whole section images were scanned with the Aperio Scanscope XT (Aperio, Vista, CA). The number of nuclei present in the epidermal layer of skin was determined using an image analysis algorithm (Spectrum Image Analysis Program, Aperio), and the number of pHH3-positive cells was counted by a pathologist blinded to the dose arm and pre-/post-treatment status of each biopsy sample (Mosaic Laboratories, Lake Forest, CA).

Disease effect endpoints were determined using Response Evaluation Criteria In Solid Tumors version 1.1. Tumour burden assessment was conducted within 21 days prior to cycle 1 day 1, every 8 weeks thereafter, and at the final visit if not performed in the past 4 weeks.

### Statistical methods

Descriptive statistics were used for demographic variables. The numbers and percentages of patients with treatment-emergent AEs (TEAEs) were tabulated using the Medical Dictionary for Regulatory Activities version 16.0 system organ class and preferred term, and further classified according to CTCAE version 4.0 criteria. The safety data set included all patients who received one or more doses of ilorasertib. The pharmacokinetic data set included all patients who received one or more doses of ilorasertib and had one or more pharmacokinetic samples. Values for pharmacokinetic parameters were determined using non-compartmental methods, with data analysed separately for each arm. A repeated measures analysis was performed for *T*_max_ and dose-normalised *C*_max_, AUC_t_, and AUC_∞_. The model included random effects for patient and fixed effects for dose level, day, and the interaction of dose level and day. To assess effects of exposure on pharmacodynamic biomarkers, *C*_max_ was employed as the directly measured marker of exposure for the rapid changes in DBP and PlGF pre-steady-state, while AUC_∞_ reflected exposure variability at the time of post-multiple-dose skin biopsy. Descriptive statistics were used to summarise ECOG performance status. The efficacy data set included all patients who received one or more doses of ilorasertib.

## Results

### Patient disposition and characteristics

A total of 58 patients were enrolled in Arms I (*n* = 23), II (*n* = 28), and III (*n* = 7). In Arm I, patients were treated at dose levels of 10 mg (*n* = 3), 20 mg (*n* = 2), 40 mg (*n* = 1), 80 mg (*n* = 3), 120 mg (*n* = 7), 140 mg (*n* = 4), and 180 mg (*n* = 3). In Arm II, patients received dose levels of 40 mg (*n* = 3), 60 mg (*n* = 3), 90 mg (*n* = 4), 130 mg (*n* = 4), 190 mg (*n* = 7), 230 mg (*n* = 4), and 340 mg (*n* = 3). In Arm III, patients were treated at 8 mg (*n* = 4), 16 mg (*n* = 1), and 32 mg (*n* = 2). All patients received one or more doses of study drug and thus comprised the intent-to-treat, efficacy, and safety datasets. Table 2 summarises patient demographics and baseline characteristics. Overall, patients were predominantly female (62%), self-reported race of ‘White’ (93%), aged < 65 years (69%), with a median age of 61 years (range: 35–81). All patients had advanced solid tumours; the most common were colorectal (24%) and ovarian (16%). Overall, 67% of patients had more than two prior drug regimens. All patients discontinued treatment, with the reasons for study drug discontinuation being radiologic evidence of progressive disease (64%), clinical evidence of progressive disease (14%), AEs (17%), and withdrawal of consent (7%).

**Table 2:** Patient demographics and baseline characteristics—intent-to-treat population

Parameter	Arm I (ilorasertib PO QD)	Arm II (ilorasertib PO BID)	Arm III (ilorasertib i.v.)	Total
	*N* = 23	*N* = 28	*N* = 7	*N *= 58
*Sex,*n* (%)*				
Female	11 (48)	20 (71)	5 (71)	36 (62)
Male	12 (52)	8 (29)	2 (29)	22 (38)
*Age, years,*n* (%)*				
<65	13 (57)	23 (82)	4 (57)	40 (69)
≥65	10 (43)	5 (18)	3 (43)	18 (31)
*Age*				
Mean (s.d.)	66 (7)	55 (11)	60 (11)	60 (11)
Median	63	55	60	61
Range	53–81	35–75	47–75	35–81
*Race,*n* (%)*				
White	21 (91)	26 (93)	7 (100)	54 (93)
Black	2 (9)	2 (7)	0	4 (7)
Ethnicity, n (%)				
Hispanic or Latino	0	3 (11)	2 (29)	5 (9)
No ethnicity	23 (100)	25 (89)	5 (71)	53 (91)
*Tumour type,*n* (%)*				
Lung	1 (4)	0	0	1 (2)
Prostate	1 (4)	0	0	1 (2)
Breast	0	2 (7)	0	2 (3)
Cervical	2 (9)	0	1 (14)	3 (5)
Pancreatic	1 (4)	5 (18)	0	6 (10)
Oesophageal	2 (9)	1 (4)	0	3 (5)
Head and neck	0	1 (4)	1 (14)	2 (3)
Ovarian	2 (9)	7 (25)	0	9 (16)
Colorectal	8 (35)	5 (18)	1 (14)	14 (24)
Other	6 (26)	7 (25)	4 (57)	17 (29)
*ECOG PS,*n* (%)*				
0	12 (52)	9 (32)	1 (14)	22 (38)
1	11 (48)	17 (61)	5 (71)	33 (57)
2	0	2 (7)	1 (14)	3 (5)
*Prior drug regimens,*n* (%)*				
0	0	1 (4)	0	1 (2)
1	4 (17)	2 (7)	1 (14)	7 (12)
2	3 (13)	6 (21)	2 (29)	11 (19)
>2	16 (70)	19 (68)	4 (57)	39 (67)

*BID* twice daily, *ECOG PS* Eastern Cooperative Oncology Group performance status, *i.v.* intravenously, *PO* orally, *QD* once daily

### Safety

TEAEs occurred in 56 of 58 (97%) patients treated with ilorasertib; grade 3 or 4 TEAEs occurred in 32 of 58 (55%) patients. The most common TEAEs reported in >20% of patients were fatigue (48%), decreased appetite (34%), hypertension (34%), nausea (26%), diarrhoea (26%), constipation (22%), and vomiting (21%). TEAEs that occurred in ≥10% of patients and grade 3 or 4 TEAEs that occurred in >2% of patients are summarised according to treatment arm in Table 3. The most common grade 3 or 4 TEAEs were hypertension (17%), diarrhoea (3%), and neutropenia (3%). Thirty-eight of 58 (66%) patients reported TEAEs that were considered possibly or probably related to ilorasertib by the investigator. The most common (≥10% of patients) treatment-related TEAEs were hypertension (29%), fatigue (24%), decreased appetite (19%), nausea (17%), diarrhoea (14%), thrombocytopenia (14%), and vomiting (10%). Sixteen (28%) patients reported grade 3 or 4 TEAEs that were considered treatment related, with the most common ( > 2% of patients) being hypertension (14%), diarrhoea (3%), and neutropenia (3%).

**Table 3:** Treatment-emergent adverse events

	Arm I (ilorasertib PO QD)	Arm II (ilorasertib PO BID)	Arm III (ilorasertib i.v.)	Total
	*N* = 23	*N* = 28	*N* = 7	*N* = 58
Any grade AE	22 (96)	27 (96)	7 (100)	56 (97)
Grade 3 or 4	14 (61)	15 (54)	3 (43)	32 (55)
*Any grade treatment-emergent adverse events reported in ≥10% of all patients*				
Fatigue	13 (57)	14 (50)	1 (14)	28 (48)
Decreased appetite	8 (35)	9 (32)	3 (43)	20 (34)
Hypertension	8 (35)	9 (32)	3 (43)	20 (34)
Diarrhoea	5 (22)	8 (29)	2 (29)	15 (26)
Nausea	8 (35)	5 (18)	2 (29)	15 (26)
Constipation	6 (26)	5 (18)	2 (29)	13 (22)
Vomiting	3 (13)	8 (29)	1 (14)	12 (21)
Urinary tract infection	2 (9)	5 (18)	2 (29)	9 (16)
Headache	5 (22)	4 (14)	0	9 (16)
Thrombocytopenia	3 (13)	5 (18)	0	8 (14)
Dyspnoea	2 (9)	3 (11)	2 (29)	7 (12)
Hypokalaemia	3 (13)	3 (11)	0	6 (10)
Hypomagnesaemia	3 (13)	3 (11)	0	6 (10)
*Grade 3 or 4 treatment-emergent adverse events reported in > 2% of all patients*				
Hypertension	4 (17)	4 (14)	2 (29)	10 (17)
Diarrhoea	1 (4)	1 (4)	0	2 (3)
Neutropenia	0	2 (7)	0	2 (3)

*AE* adverse event, *BID* twice daily, *i.v.* intravenously, *PO* orally, *QD* once daily

Treatment-emergent serious AEs were reported in 21 (36%) patients, with events occurring in >2% of patients including small intestinal obstruction (7%) and fatigue (3%). TEAEs that led to discontinuation were reported in 10 (17%) patients. However, none of these events, including hypertension, fatigue, and specific gastrointestinal events, occurred in more than one patient. TEAEs resulted in deaths of three patients, including one patient in Arm I with renal failure, and two patients in Arm II, one each with pulmonary embolism and progression of a malignant neoplasm. All three deaths occurred within 30 days of the patients discontinuing the study. Nevertheless, none of the deaths were considered related to ilorasertib by the investigator, but rather to the underlying disease or disease progression.

Six (10%) patients experienced a total of ten DLTs. In Arm I, DLTs were grade 3 abdominal pain and grade 3 fatigue, occurring in one patient each, treated at the 120-mg and 180-mg dose levels, respectively. Another patient (180 mg) had grade 3 hypertension and diarrhoea. In Arm II, DLTs were grade 3 posterior reversible encephalopathy syndrome and grade 3 leucopenia in one patient each, treated at 190-mg and 340-mg dose levels, respectively. A further patient in Arm II (340 mg) experienced grade 4 hyperuricaemia, grade 3 non-cardiac chest pain, grade 3 dehydration, and grade 3 stress-induced cardiomyopathy. In Arms I and II, ilorasertib at 180 mg QD and 340 mg BID, respectively, met the protocol criteria for exceeding the MTD. No patient in Arm III had a DLT. The sponsor’s strategic and resource allocation decisions led to study termination without establishment of the MTD. Two investigator-sponsored trials (NCT02478320 and NCT02540876), based on the findings reported in this manuscript, were ongoing at the time of manuscript submission.

### Pharmacokinetics

**Fig. 1:**
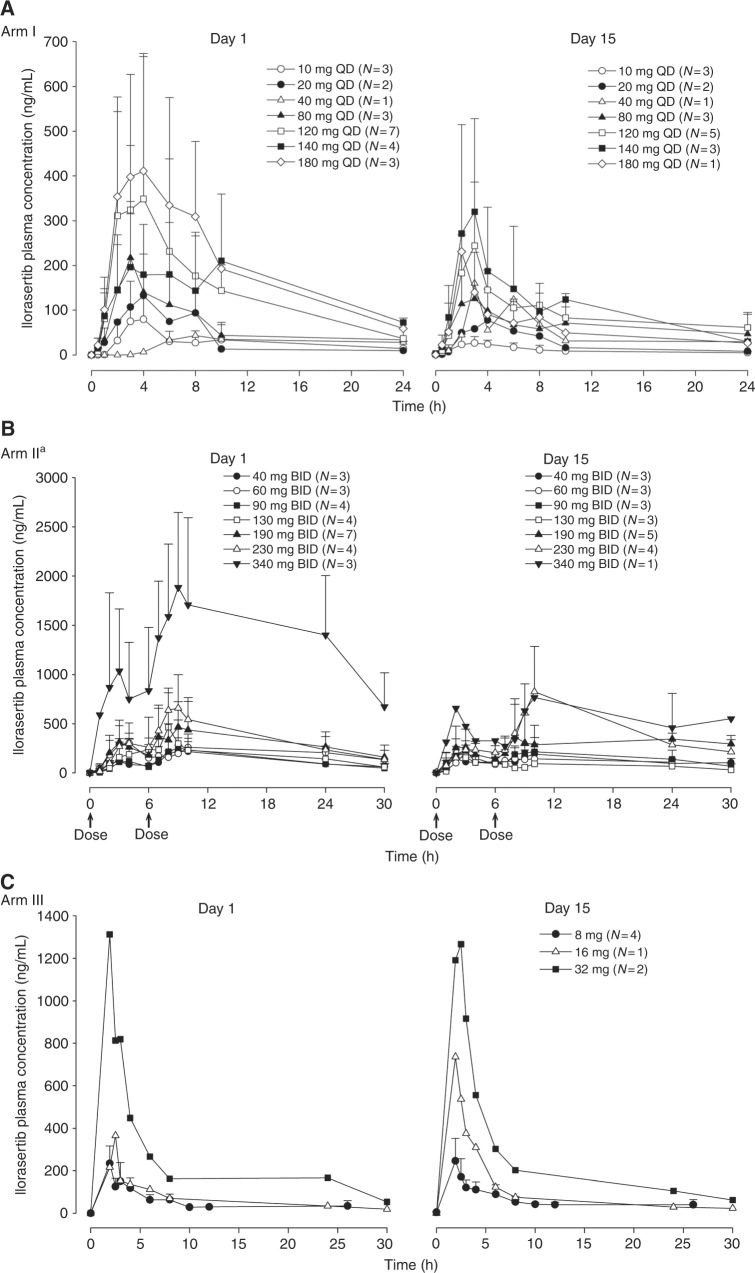
Ilorasertib plasma concentration-time profiles (day 1 and 15). Mean (+s.d.) ilorasertib plasma concentration-time profiles (linear scale) following oral administration in Arm I (**a**), Arm II (**b**), and i.v. infusion in Arm III (**c**). ^a^In the 340-mg dose cohort, two of three patients had high ilorasertib exposure at day 1; however, there were no available data for these two patients at day 15. *BID* twice daily; *i.v.* intravenous; *QD* once daily

Ilorasertib plasma concentration-time profiles following QD PO administration in Arm I, BID PO administration in Arm II, and QD i.v. administration in Arm III are summarised in Fig. 1 for days 1 and 15 of cycle 1. Plasma concentrations of ilorasertib in Arm I peaked at ~4 h after a single dose under fasting conditions across dose levels. In Arm II, with oral administration in two divided doses, *T*_max_ was delayed by several hours, whereas in Arm III, with i.v. administration, *T*_max_ was 2 h (end of infusion). The mean *t*_1/2_ after a single dose of ilorasertib was ~12 h. Ilorasertib exposures, as assessed by *C*_max_ and AUC, were approximately dose proportional over a dose range of 10–180 mg QD in Arm I, 40–340 mg BID in Arm II, and 8–32 mg i.v. in Arm III. The mean (±s.d.) pharmacokinetic parameters of ilorasertib following QD PO administration in Arm I on days 1 and 15 of cycle 1 are summarised in Supplementary Table S1 and Supplementary Table S2, respectively. The respective data for Arms II and III are summarised in Supplementary Tables S3–S6.

### Pharmacodynamics

**Fig. 2:**
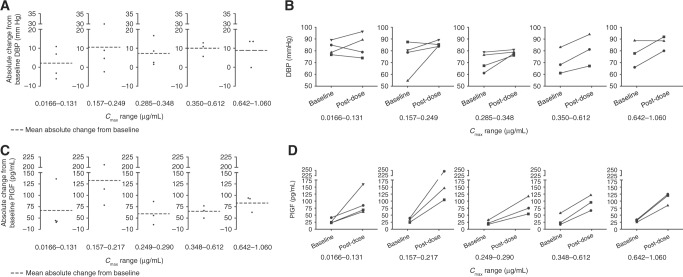
Changes from baseline in DBP (**a**, **b**) and PlGF (**c**,** d**) following ilorasertib administration. *C*_max_, maximum observed plasma concentration, *DBP* diastolic blood pressure, *PlGF* platelet-derived growth factor

**Fig. 3:**
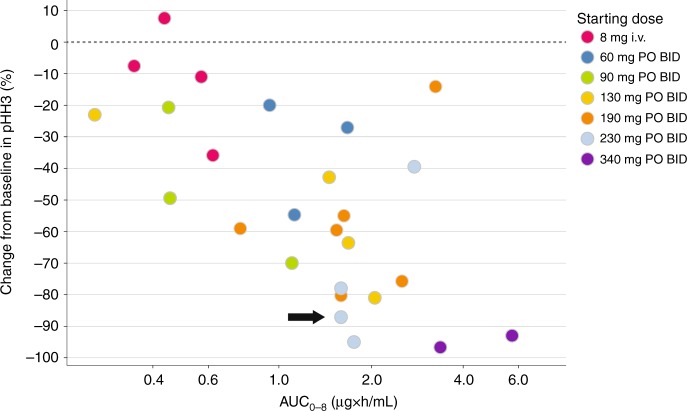
Reduction in skin tissue (epidermis) pHH3 is associated with ilorasertib exposure. pHH3-positive cells (%) = number of pHH3-positive cells/total number of cells enumerated. Change from baseline in pHH3 (%) = [pHH3-positive cells at post-dose (%)/pHH3-positive cells at pre-dose (%)]×100. One responder is indicated by the arrow; for the other responder, pHH3 data were not available. *AUC*_*0-8*_ area under the concentration-time curve from time 0 to 8 h, *BID* twice daily, i.v. intravenously, *pHH3* phosphohistone H3, *PO* orally

Changes from baseline in DBP and PlGF following ilorasertib administration are shown in Fig. 2. DBP elevation^21–23^ (Fig. 2a, b) and increase in PlGF^24–26^ (Fig. 2c, d) reflect VEGFR2 inhibition. Increases in DBP and PlGF did not seem to be dose proportional. Based on skin biopsy samples from patients treated with ilorasertib, increasing exposure to ilorasertib resulted in dose-dependent decreases of pHH3, indicating inhibition of Aurora kinase B activity in the epidermis (Fig. 3). The maximum VEGFR inhibition was reached at lower doses and systemic concentrations than the Aurora inhibitory effects. Peak changes in DBP and PlGF were observed in the second quintile of exposure, whereas maximum pHH3 inhibition was observed at the highest exposure.

### Therapeutic activity

Two patients in Arm II had a partial response as their best response. Both patients had received two or more prior drug regimens. One of the responders (duration of response: 405 days) had skin cancer (basal cell carcinoma) and was treated with ilorasertib at dose of 40 mg BID. The other responder (duration of response: 168 days), with an adenocarcinoma of unknown primary site, received ilorasertib at 230 mg BID.

## Discussion

Pharmacodynamic biomarker/pharmacokinetic analysis revealed clinically relevant differences in the exposure/response relationships for the two intended targets of the multikinase inhibitor ilorasertib. Peak inhibition of VEGFR2 was evident by increases in DBP and plasma PlGF concentrations in the second plasma exposure quintile of ilorasertib, whereas the marker of Aurora kinase B inhibition (percentage change from baseline in pHH3 in skin biopsies) reached peak effect only in the two highest quintiles. These observations have informed the further development of ilorasertib and illuminate considerations in the development of other multikinase inhibitors.

Clinical development of novel anticancer therapeutics often assumes that the dose-limiting AEs will be mechanism-dependent events, directly related to engagement of the intended primary target of the drug. In the case of compounds that inhibit multiple targets, intensive scrutiny of the pharmacokinetic/pharmacodynamic relationships would be necessary for optimal development of the compound. We observed in this cohort of solid tumour patients that complete VEGFR2 inhibition was achieved at lower ilorasertib exposure, while maximum Aurora kinase B inhibition effects in tissue required higher exposures. These findings are consistent with cellular assays that revealed ilorasertib inhibition of VEGFR2 to be two- to five-fold more potent than for Aurora kinase B.^10^ But this differential in in vitro potency is unlikely to be the exclusive predictive factor of differential pharmacodynamic effects. For example, the primary site of VEGFR2 expression is in systemic endothelium, a compartment that is more rapidly and completely accessed by ilorasertib than tissue Aurora kinase B. In Arm I of this study, dose-escalation was discontinued based, in part, on development of grade 3 hypertension by one of the patients at the 180-mg dose. In the earlier study conducted in patients with haematologic malignancies,^15^ the DLTs were those previously associated with VEGFR2 kinase inhibitors: hypertension and pancreatitis, but detected at higher doses than in this solid tumour patient investigation. In contrast, the AE most likely to be caused by Aurora kinase inhibition, leucopenia, was routinely observed in both the haematologic and solid malignancy trials, but was not dose limiting.

These observations describe a primary challenge to the further development of ilorasertib as a multikinase inhibitor—inhibition of the intended targets typically occurs at different systemic concentrations, and toxic effects of VEGFR2 inhibition may be encountered by some patients before the potentially therapeutic effects of Aurora kinase inhibition are reached. However, differences among patients in intrinsic sensitivity to the pharmacodynamic effects of VEGFR2 inhibition have been well described.^21,27^ One potential strategy is to exclude patients who are intrinsically sensitive to low-exposure VEGFR2 inhibition and associated AEs, and administer full-dose ilorasertib exclusively to patients who tolerate maximum inhibition of VEGFR2. An alternative strategy would be to identify a predictive biomarker that predisposes tumours to greater sensitivity to Aurora kinase inhibition at achievable exposures. A clinical investigation based on the latter strategy is ongoing (NCT02540876).

These observations are based on retrospective analysis of prospectively collected data, but the study was not initially designed to perform this analysis. Complete data were not available for all patients, so these results could be biased in unappreciated ways. Although the blood pressure and plasma PlGF measurements were obtained with validated methods, these biomarkers of VEGFR2 inhibition have not yet been clinically qualified as measures of VEGFR2 kinase inhibition. The skin pHH3 assay employed in this study is also analytically validated and, although not functionally validated as a biomarker of pharmacologic inhibition of Aurora kinases, pHH3 inhibition has also been observed with other Aurora kinase inhibitors.^28,29^ Furthermore, although pHH3 inhibition may be observed in normal cells, this occurs at a reduced degree compared with highly proliferating tumour cells. We considered these reported biomarkers to be fit-for-purpose in the context of this study, but acknowledge these points and have considered them in our analysis and conclusions.

The current study confirmed previous phase 1 tolerability and safety findings of ilorasertib. Treatment of advanced solid tumour patients with ilorasertib resulted in engagement of the intended targets, but the pharmacokinetic/pharmacodynamics analyses revealed differential effects on these targets. The future development of multitargeted agents could benefit from systematic incorporation of similar pharmacokinetic/fit-for-purpose pharmacodynamic biomarker analyses into early stage investigations.

## Electronic supplementary material

Supplementary Table S1(DOCX 26 kb)

Supplementary Table S2(DOCX 26 kb)

Supplementary Table S3(DOCX 26 kb)

Supplementary Table S4(DOCX 26 kb)

Supplementary Table S5(DOCX 24 kb)

Supplementary Table S6(DOCX 24 kb)
