# Supplementary material for: Clinical pharmacodynamic/exposure characterisation of the multikinase inhibitor ilorasertib (ABT-348) in a phase 1 dose-escalation trial
Source: Br J Cancer. 2018 Mar 19;118(8):1042–50. doi: 10.1038/s41416-018-0020-2 (PMC5931107; doi:10.1038/s41416-018-0020-2)
Supplement: Supplementary file 1 — Supplementary Table S1(DOCX 26 kb) [file 41416_2018_20_MOESM1_ESM.docx]

| **Supplementary Table S1: Mean (± SD) pharmacokinetic parameters of ilorasertib following oral QD administration, Arm I (day 1)** | | | | | | | | |
| --- | --- | --- | --- | --- | --- | --- | --- | --- |
| **Pharmacokinetic parameter** (**units)** | **Ilorasertib dose (mg)** | | | | | | | |
|  | **10** | **20** | **40** | **80** | **120** | **140** | **180** | **All** |
| N | 3 | 2^a^ | 1 | 3 | 7 | 4 | 3 | 23 |
| t_1/2_ (h)^b^ | 13.0 ± 0.4 | 15.0 (10.5, 26.1) | ND | 16.0 ± 1.8 | 9.9 ± 7.2^c^ | 15.8 ± 23.6 | 9.5 ± 2.7 | 12.2 ± 6.9^d^ |
| T_max_ (h) | 3.7 ± 0.6 | 5.0 (4.0, 6.0) | 8.0 | 3.0 ± 0.0 | 3.8 ± 2.2 | 4.8 ± 1.5 | 4.0 ± 2.0 | 4.2 ± 1.8 |
| C_max_ (μg/mL) | 0.08 ± 0.10 | 0.13 (0.25, 0.02) | 0.04 | 0.22 ± 0.10 | 0.49 ± 0.33 | 0.24 ± 0.13 | 0.49 ± 0.24 | ND |
| AUC_t_ (μg•h/mL) | 0.68 ± 0.68 | 1.76 (3.23, 0.29) | 0.64 | 1.90 ± 1.10 | 3.60 ± 2.50 | 3.08 ± 1.58 | 6.24 ± 3.43 | ND |
| AUC_∞_ (μg•h/mL) | 0.91 ± 0.91 | 2.26 (3.87, 0.66) | ND | 2.82 ± 1.54 | 5.40 ± 2.62^c^ | 5.40 ± 1.80 | 7.89 ± 5.22 | ND |
| C_max_/dose (ng/mL/mg) | 8.1 ± 9.6 | 6.6 (12.5, 0.83) | 1.1 | 2.7 ± 1.2 | 4.1 ± 2.8 | 1.7 ± 0.9 | 2.7 ± 1.3 | 3.9 ± 4.3 |
| AUC_t_/dose (ng•h/mL/mg) | 68.0 ± 68.3 | 87.9 (162, 14.4) | 16.1 | 23.7 ± 13.8 | 30.0 ± 20.9 | 22.0 ± 11.3 | 34.7± 19.1 | 37.8 ± 39.6 |
| AUC_∞_/dose (ng•h/mL/mg) | 91.5 ± 90.6 | 113 (193, 32.9) | ND | 35.3 ± 19.3 | 45.0 ± 21.8^c^ | 38.5 ± 12.9 | 43.8 ± 29.0 | 55.3 ± 49.3^d^ |
| CL/F (L/h) | 24.7 ± 25.1 | 17.8 (5.2, 30.4) | ND | 37.0 ± 24.6 | 25.7 ± 9.3^c^ | 28.1 ± 8.8 | 29.4 ± 15.5 | 27.4 ± 14.9^d^ |
| Abbreviations: AUC_∞_, area under the plasma concentration-time curve from time 0 to infinity; AUC_t_ area under the plasma concentration-time curve from time 0 to time of last measurable concentration; CL/F, apparent oral clearance; C_max_, maximum observed plasma concentration; ND, not determined; QD, once daily; SD, standard deviation; t_1/2,_ terminal phase elimination half-life; T_max_, time to C_max_.  ^a^N = 2; parameters reported as mean (individual parameters).  ^b^Harmonic mean and pseudo SD.  ^c^N = 6.  ^d^N = 21. | | | | | | | | |
