# Supplementary material for: Clinical pharmacodynamic/exposure characterisation of the multikinase inhibitor ilorasertib (ABT-348) in a phase 1 dose-escalation trial
Source: Br J Cancer. 2018 Mar 19;118(8):1042–50. doi: 10.1038/s41416-018-0020-2 (PMC5931107; doi:10.1038/s41416-018-0020-2)
Supplement: Supplementary file 2 — Supplementary Table S2(DOCX 26 kb) [file 41416_2018_20_MOESM2_ESM.docx]

| **Supplementary Table S2: Mean (± SD) pharmacokinetic parameters of ilorasertib following oral QD administration, Arm I (day 15)** | | | | | | | | |
| --- | --- | --- | --- | --- | --- | --- | --- | --- |
| **Pharmacokinetic parameter** (**units)** | **Ilorasertib dose (mg)** | | | | | | | |
|  | **10** | **20** | **40** | **80** | **120** | **140** | **180** | **All** |
| N | 3 | 2^a^ | 1 | 3 | 5 | 3 | 1 | 18 |
| t_1/2_ (h)^b^ | 11.7 ± 1.4 | 16.5 (16.2, 16.7) | 11.5 | 16.8 ± 2.8 | 18.2 ± 7.2^c^ | 10.1 (11.5, 9.1)^a^ | 11.6 | 14.0 ± 4.0^d^ |
| T_max_ (h) | 3.0 ± 1.0 | 5.0 (4.0, 6.0) | 3.0 | 4.0 ± 2.0 | 4.1 ± 2.2 | 10.1 ± 12.3 | 2.0 | 4.8 ± 5.1 |
| C_max_ (μg/mL) | 0.03 ± 0.02 | 0.08 (0.14, 0.02) | 0.16 | 0.13 ± 0.10 | 0.25 ± 0.14 | 0.33 ± 0.19 | 0.23 | ND |
| AUC_t_ (μg•h/mL) | 0.30 ± 0.16 | 1.01 (1.72, 0.31) | 1.15 | 1.59 ± 1.04 | 2.25 ± 0.61 | 3.08 ± 1.02 | 1.46 | ND |
| AUC_∞_ (μg•h/mL) | 0.38 ± 0.20 | 1.46 (2.41, 0.51) | 1.64 | 2.75 ± 2.15 | 4.31 ± 2.38^c^ | 4.13 (4.96, 3.31)^a^ | 1.91 | ND |
| C_max_/dose (ng/mL/mg) | 3.3 ± 1.6 | 4.0 (7.1, 0.89) | 4.0 | 1.6 ± 1.3 | 2.1 ± 1.1 | 2.4 ± 1.4 | 1.3 | 2.5 ± 1.7 |
| AUC_t_/dose (ng•h/mL/mg) | 29.9 ± 16.4 | 50.7 (86.0, 15.3) | 28.7 | 19.9 ± 13.0 | 18.8 ± 5.1 | 22.0 ± 7.3 | 8.1 | 24.9 ± 18.1 |
| AUC_∞_/dose (ng•h/mL/mg) | 38.2 ± 19.7 | 73.0 (120, 25.5) | 41.0 | 34.3 ± 26.9 | 35.9 ± 19.9^c^ | 29.5 (35.4, 23.7)^a^ | 10.6 | 38.6 ± 27.5^d^ |
| CL/F (L/h) | 31.8 ± 17.2 | 23.8 (8.3, 39.2) | 24.4 | 48.0 ± 40.6 | 33.7 ± 15.0^c^ | 35.3 (28.3, 42.3)^a^ | 94.4 | 38.2 ± 25.0^d^ |
| Abbreviations: AUC_∞_, area under the plasma concentration-time curve from time 0 to infinity; AUC_t_ area under the plasma concentration-time curve from time zero to time of last measurable concentration; CL/F, apparent oral clearance; C_max_, maximum observed plasma concentration; ND, not determined; QD, once daily; SD, standard deviation; t_1/2,_ terminal phase elimination half-life; T_max_, time to C_max_.  ^a^N = 2, parameters reported as mean (individual parameters).  ^b^Harmonic mean and pseudo SD.  ^c^N = 4.  ^d^N = 16. | | | | | | | | |
