# Supplementary material for: Clinical pharmacodynamic/exposure characterisation of the multikinase inhibitor ilorasertib (ABT-348) in a phase 1 dose-escalation trial
Source: Br J Cancer. 2018 Mar 19;118(8):1042–50. doi: 10.1038/s41416-018-0020-2 (PMC5931107; doi:10.1038/s41416-018-0020-2)
Supplement: Supplementary file 3 — Supplementary Table S3(DOCX 26 kb) [file 41416_2018_20_MOESM3_ESM.docx]

| **Supplementary Table S3: Mean (± SD) pharmacokinetic parameters of ilorasertib following oral BID administration, Arm II (day 1)** | | | | | | | | |
| --- | --- | --- | --- | --- | --- | --- | --- | --- |
| **Pharmacokinetic parameter (unit)** | **Ilorasertib dose (mg)** | | | | | | | |
|  | **40** | **60** | **90** | **130** | **190** | **230** | **340** | **All** |
| N | 3 | 3 | 3 | 4 | 7 | 3 | 3 | 26 |
| t_1/2_ (h)^a^ | 11.0 (16.3, 8.3)^b^ | ND | 10.1 (9.5, 10.9)^b^ | 18.6 ± 17.6^c^ | 13.7 ± 9.0^d^ | 9.0 (9.3, 8.7)^b^ | 39.6^e^ | 13.0 ± 6.2^f^ |
| T_max_ (h) | 7.3 ± 3.8 | 5.7 ± 3.8 | 9.0 ± 1.0 | 7.5 ± 1.7 | 10.2 ± 6.7 | 8.7 ± 1.2 | 15.0 ± 8.0 | 9.2 ± 5.0 |
| C_max_ (μg/mL) | 0.25 ± 0.12 | 0.38 ± 0.14 | 0.30 ± 0.1 | 0.60 ± 0.39 | 0.54 ± 0.28 | 0.70 ± 0.23 | 2.04 ± 0.87 | ND |
| AUC_t_ (μg•h/mL) | 3.72 ± 1.64 | 5.45 ± 1.46 | 3.92 ± 1.53 | 5.07 ± 3.18 | 8.10 ± 4.44 | 10.1 ± 3.62 | 39.3 ± 14.8 | ND |
| AUC_∞_ (μg•h/mL) | 3.99 (2.85, 5.12)^b^ | ND | 4.03 (2.55, 5.52)^b^ | 6.53 ± 4.46^c^ | 17.8 ± 11.3^d^ | 9.14 (8.31, 9.97)^b^ | 98.8^e^ | ND |
| C_max_/dose (ng/mL/mg) | 3.1 ± 1.5 | 3.2 ± 1.2 | 1.7 ± 0.6 | 2.3 ± 1.5 | 1.4 ± 0.8 | 1.5 ± 0.5 | 3.0 ± 1.3 | 2.2 ± 1.2 |
| AUC_t_/dose (ng•h/mL/mg) | 46.5 ± 20.5 | 45.4 ± 12.2 | 21.8 ± 8.5 | 19.5 ± 12.2 | 21.3 ± 11.7 | 21.9 ± 7.9 | 57.8 ± 21.8 | 31.1 ± 18.8 |
| AUC_∞_/dose (ng•h/mL/mg) | 49.9 (35.6, 64.1)^b^ | ND | 22.4 (14.1, 30.6)^b^ | 25.1 ± 17.2^c^ | 46.8 ± 29.7^d^ | 19.9 (18.1, 21.7)^b^ | 145^e^ | 42.3 ± 36.5^f^ |
| CL/F (L/h) | 21.8 (28.1, 15.6)^b^ | ND | 51.7 (70.7, 32.6)^b^ | 51.5 ± 25.9^c^ | 29.6 ± 17.9^d^ | 50.8 (55.4, 46.1)^b^ | 6.9^e^ | 37.7 ± 21.8^f^ |
| Abbreviations: AUC_∞_, area under the plasma concentration-time curve from time 0 to infinity; AUC_t_ area under the plasma concentration-time curve from time zero to time of last measurable concentration; BID, twice daily;  CL/F, apparent oral clearance; C_max_, maximum observed plasma concentration; ND, not determined; SD, standard deviation; t_1/2,_ terminal phase elimination half-life; T_max_, time to C_max_.  ^a^Harmonic mean and pseudo SD.  ^b^N = 2; parameters reported as mean (individual parameters).  ^c^N = 3.  ^d^N = 4.  ^e^N = 1; parameters reported as individual value.  ^f^N = 14. | | | | | | | | |
