# Supplementary material for: Clinical pharmacodynamic/exposure characterisation of the multikinase inhibitor ilorasertib (ABT-348) in a phase 1 dose-escalation trial
Source: Br J Cancer. 2018 Mar 19;118(8):1042–50. doi: 10.1038/s41416-018-0020-2 (PMC5931107; doi:10.1038/s41416-018-0020-2)
Supplement: Supplementary file 4 — Supplementary Table S4(DOCX 26 kb) [file 41416_2018_20_MOESM4_ESM.docx]

| **Supplementary Table S4: Mean (± SD) pharmacokinetic parameters of ilorasertib following oral BID administration, Arm II (day 15)** | | | | | | | | |
| --- | --- | --- | --- | --- | --- | --- | --- | --- |
| **Pharmacokinetic parameter (units)** | **Ilorasertib dose (mg)** | | | | | | | |
|  | **40** | **60** | **90** | **130** | **190** | **230** | **340** | **All** |
| N | 3 | 3 | 3 | 3 | 5 | 4 | 1 | 22 |
| t_1/2_ (h)^a^ | ND | 11.8^b^ | 16.2 (23.4, 12.4)^c^ | 7.5^b^ | 8.4 (6.6, 11.3)^c^ | 8.0^b^ | ND | 9.9 ± 3.7^d^ |
| T_max_ (h) | 10.0 ± 0.0 | 4.8 ± 2.8 | 7.6 ± 5.4 | 11.0 ± 11.5 | 13.2 ± 10.2 | 9.5 ± 0.6 | 10.0 | 9.7 ± 6.6 |
| C_max_ (μg/mL) | 0.19 ± 0.11 | 0.23 ± 0.06 | 0.32 ± 0.10 | 0.26 ± 0.28 | 0.63 ± 0.38 | 0.79 ± 0.46 | 0.77 | ND |
| AUC_t_ (μg•h/mL) | 3.75 ± 2.05 | 2.92 ± 0.71 | 4.06 ± 1.51 | 2.40 ± 1.29 | 6.69 ± 5.22 | 11.2 ± 4.34 | 15.7 | ND |
| AUC_∞_ (μg•h/mL) | ND | 5.43^b^ | 5.33 (7.41, 3.26)^c^ | 3.96^b^ | 10.7 (7.94, 13.4)^c^ | 13.0^b^ | ND | ND |
| C_max_/dose (ng/mL/mg) | 2.4 ± 1.3 | 1.9 ± 0.47 | 1.8 ± 0.54 | 1.0 ± 1.1 | 1.7 ± 1.0 | 1.7 ± 1.0 | 1.1 | 1.7 ± 0.9 |
| AUC_t_/dose (ng•h/mL/mg) | 46.9 ± 25.6 | 24.3 ± 5.9 | 22.5 ± 8.4 | 9.2 ± 5.0 | 17.6 ± 13.7 | 24.4 ± 9.4 | 23.0 | 23.5 ± 15.5 |
| AUC_∞_/dose (ng•h/mL/mg) | ND | 45.3^b^ | 29.6 (41.1, 18.1)^c^ | 15.2^b^ | 28.0 (20.9, 35.2)^c^ | 28.2^b^ | ND | 29.1 ± 11.7^d^ |
| CL/F (L/h) | ND | 22.1^b^ | 39.8 (24.3, 55.2)^c^ | 65.7^b^ | 38.1 (47.8, 28.4)^c^ | 35.5^b^ | ND | 39.9 ± 16.7^d^ |
| Abbreviations: AUC_∞_, area under the plasma concentration-time curve from time 0 to infinity; AUC_t_ area under the plasma concentration-time curve from time zero to time of last measurable concentration; BID, twice daily; CL/F, apparent oral clearance; C_max_, maximum observed plasma concentration; ND, not determined; SD, standard deviation; t_1/2,_ terminal phase elimination half-life; T_max_, time to C_max_.  ^a^Harmonic mean and pseudo SD.  ^b^N = 1; parameters reported as individual value.  ^c^N = 2; parameters reported as mean (individual parameters).  ^d^N = 7. | | | | | | | | |
