# Supplementary material for: Clinical pharmacodynamic/exposure characterisation of the multikinase inhibitor ilorasertib (ABT-348) in a phase 1 dose-escalation trial
Source: Br J Cancer. 2018 Mar 19;118(8):1042–50. doi: 10.1038/s41416-018-0020-2 (PMC5931107; doi:10.1038/s41416-018-0020-2)
Supplement: Supplementary file 5 — Supplementary Table S5(DOCX 24 kb) [file 41416_2018_20_MOESM5_ESM.docx]

| **Supplementary Table S5: Mean (± SD) pharmacokinetic parameters of ilorasertib following i.v. infusion, Arm III (day 1)** | | | | |
| --- | --- | --- | --- | --- |
| **Pharmacokinetic parameter (units)** | **Ilorasertib dose (mg)** | | | |
|  | **8** | **16** | **32** | **All** |
| N | 4 | 1 | 2^c^ | 7 |
| t_1/2_ (h)^a^ | 8.5 ± 4.1^b^ | 10.7 | 9.3 (9.1, 9.5) | 9.1 ± 3.3^d^ |
| T_max_ (h) | 2.0 ± 0.2 | 2.5 | 1.9 (1.9, 1.9) | 2.0 ± 0.2 |
| C_max_ (μg/mL) | 0.23 ± 0.08 | 0.37 | 1.31 (1.69, 0.94) | ND |
| AUC_t_ (μg•h/mL) | 1.48 ± 0.34 | 1.91 | 8.46 (12.5, 4.45) | ND |
| AUC_∞_ (μg•h/mL) | 2.19 ± 0.31^b^ | 2.44 | 9.18 (13.6, 4.80) | ND |
| C_max_/dose (ng/mL/mg) | 29.3 ± 10.3 | 22.9 | 41.0 (52.8, 29.2) | 31.8 ± 12.0 |
| AUC_t_/dose (ng•h/mL/mg) | 185 ± 42.9 | 119 | 265 (390, 139) | 198 ± 93.7 |
| AUC_∞_/dose (ng•h/mL/mg) | 273 ± 38.5^b^ | 152 | 287 (424, 150) | 258 ± 104^d^ |
| CL (L/h) | 3.71 ± 0.49^b^ | 6.56 | 4.51 (2.36, 6.66) | 4.45 ± 1.78^d^ |
| Abbreviations: AUC_∞_, area under the plasma concentration-time curve from time 0 to infinity; AUC_t_ area under the plasma concentration-time curve from time zero to time of last measurable concentration; CL, clearance; C_max_, maximum observed plasma concentration; i.v., intravenous; ND, not determined; SD, standard deviation; t_1/2,_ terminal phase elimination half-life; T_max_, time to C_max_.  ^a^Harmonic mean and pseudo SD.  ^b^N = 3.  ^c^Parameters reported as mean (individual parameters).  ^d^N = 6. | | | | |
