# Supplementary material for: Clinical pharmacodynamic/exposure characterisation of the multikinase inhibitor ilorasertib (ABT-348) in a phase 1 dose-escalation trial
Source: Br J Cancer. 2018 Mar 19;118(8):1042–50. doi: 10.1038/s41416-018-0020-2 (PMC5931107; doi:10.1038/s41416-018-0020-2)
Supplement: Supplementary file 6 — Supplementary Table S6(DOCX 24 kb) [file 41416_2018_20_MOESM6_ESM.docx]

| **Supplementary Table S6: Mean (± SD) pharmacokinetic parameters of ilorasertib following i.v. infusion, Arm III (day 15)** | | | | |
| --- | --- | --- | --- | --- |
| **Pharmacokinetic parameter (units)** | **Ilorasertib dose (mg)** | | | |
|  | **8** | **16** | **32** | **All** |
| N | 4 | 1 | 2^c^ | 7 |
| t_1/2_ (h)^a^ | 14.0 ± 9.7^b^ | 9.6 | 11.3 (13.1, 9.9) | 12.1 ± 4.4^d^ |
| T_max_ (h) | 1.9 ± 0.0 | 1.9 | 2.2 (2.5, 1.9) | 2.0 ± 0.2 |
| C_max_ (μg/mL) | 0.25 ± 0.11 | 0.74 | 1.37 (1.85, 0.88) | ND |
| AUC_t_ (μg•h/mL) | 1.75 ± 0.57 | 3.04 | 7.30 (9.71, 4.88) | ND |
| AUC_∞_ (μg•h/mL) | 2.88 ± 1.99^b^ | 3.45 | 8.41 (11.4, 5.37) | ND |
| C_max_/dose (ng/mL/mg) | 30.8 ± 13.3 | 46 | 42.7 (57.8, 27.6) | 36.4 ± 14.7 |
| AUC_t_/dose (ng•h/mL/mg) | 219 ± 71.5 | 190 | 228 (304, 153) | 217 ± 68.0 |
| AUC_∞_/dose (ng•h/mL/mg) | 360 ± 249^b^ | 215 | 263 (358, 168) | 303 ± 180^d^ |
| CL (L/h) | 3.61 ± 1.82^b^ | 4.64 | 4.38 (2.80, 5.96) | 4.04 ± 1.60^d^ |
| Abbreviations: AUC_∞_, area under the plasma concentration-time curve from time 0 to infinity; AUC_t_ area under the plasma concentration-time curve from time zero to time of last measurable concentration; CL, clearance; C_max_, maximum observed plasma concentration; i.v., intravenous; ND, not determined; SD, standard deviation; t_1/2,_ terminal phase elimination half-life; T_max_, time to C_max_.  ^a^Harmonic mean and pseudo SD.  ^b^N = 3.  ^c^Parameters reported as mean (individual parameters).  ^d^N = 6. | | | | |
